# Cardiac ischemia in patients with septic shock randomized to vasopressin or norepinephrine

**DOI:** 10.1186/cc12789

**Published:** 2013-06-20

**Authors:** Sangeeta Mehta, John Granton, Anthony C Gordon, Deborah J Cook, Stephen Lapinsky, Gary Newton, Kris Bandayrel, Anjuli Little, Chuin Siau, Dieter Ayers, Joel Singer, Terry CK Lee, Keith R Walley, Michelle Storms, D James Cooper, Cheryl L Holmes, Paul Hebert, Jeffrey Presneill, James A Russell

**Affiliations:** 1 Department of Medicine and Interdepartmental Division of Critical Care Medicine, Mount Sinai Hospital, University of Toronto, Toronto, Ontario, Canada; 2 University Health Network-General Division, University of Toronto, Toronto, Ontario, Canada; 3 Intensive Care, Charing Cross Hospital, Imperial College, London, UK; 4 Departments of Medicine, Clinical Epidemiology & Biostatistics, McMaster University, Hamilton, Ontario, Canada; 5 Department of Medicine and Division of Cardiology, Mount Sinai Hospital, University of Toronto, Toronto, Ontario, Canada; 6 Department of Respiratory Medicine, Changi General Hospital, Singapore; 7 School of Population and Public Health, Centre for Health Evaluation and Outcome Sciences, and University of British Columbia, Vancouver, British Columbia, Canada; 8 Critical Care Research Laboratories, Heart and Lung Institute, St. Paul's Hospital, and University of British Columbia, Vancouver, British Columbia, Canada; 9 Alfred Hospital, Monash University, Melbourne, Australia; 10 Kelowna General Hospital, Kelowna, and University of British Columbia, Kelowna, British Columbia, Canada; 11 Ottawa Hospital-General Campus, and University of Ottawa, Ottawa, Ontario, Canada; 12 Mater Health Services, University of Queensland and Monash University, South Brisbane Queensland, Australia

**Keywords:** Septic shock, myocardial ischemia, vasopressin, norepinephrine, troponin, electrocardiogram

## Abstract

**Introduction:**

Cardiac troponins are sensitive and specific biomarkers of myocardial necrosis. We evaluated troponin, CK, and ECG abnormalities in patients with septic shock and compared the effect of vasopressin (VP) versus norepinephrine (NE) on troponin, CK, and ECGs.

**Methods:**

This was a prospective substudy of a randomized trial. Adults with septic shock randomly received, blinded, a low-dose infusion of VP (0.01 to 0.03 U/min) or NE (5 to 15 μg/min) in addition to open-label vasopressors, titrated to maintain a mean blood pressure of 65 to 75 mm Hg. Troponin I/T, CK, and CK-MB were measured, and 12-lead ECGs were recorded before study drug, and 6 hours, 2 days, and 4 days after study-drug initiation. Two physician readers, blinded to patient data and drug, independently interpreted ECGs.

**Results:**

We enrolled 121 patients (median age, 63.9 years (interquartile range (IQR), 51.1 to 75.3), mean APACHE II 28.6 (SD 7.7)): 65 in the VP group and 56 in the NE group. At the four time points, 26%, 36%, 32%, and 21% of patients had troponin elevations, respectively. Baseline characteristics and outcomes were similar between patients with positive versus negative troponin levels. Troponin and CK levels and rates of ischemic ECG changes were similar in the VP and the NE groups. In multivariable analysis, only APACHE II was associated with 28-day mortality (OR, 1.07; 95% CI, 1.01 to 1.14; *P *= 0.033).

**Conclusions:**

Troponin elevation is common in adults with septic shock. We observed no significant differences in troponin, CK, and ECGs in patients treated with vasopressin and norepinephrine. Troponin elevation was not an independent predictor of mortality.

**Trial registration:**

Controlled-trials.com ISRCTN94845869

## Introduction

Cardiac ischemia occurs frequently in critically ill patients and is associated with increased mortality [1]. Given that most critically ill patients cannot communicate symptoms, the diagnosis of cardiac ischemia can be challenging and necessitates that clinicians rely on cardiac biomarkers and electrocardiograms (ECGs). Patients receiving vasopressors may be at even higher risk of cardiac ischemia than other patients because of coronary artery vasoconstriction, increased systemic vasoconstriction-induced afterload [2], or catecholamine-driven increases in myocardial oxygen demand.

The Vasopressin in Severe Sepsis Trial (VASST) randomized patients with septic shock to vasopressin or norepinephrine [3]. Overall, no significant difference occurred in the primary outcome (28-day mortality) between the vasopressin and the norepinephrine groups (35.4% versus 39.3%, respectively; *P *= 0.26). Further, no differences were found in the clinical diagnosis of myocardial infarction/ischemia in the two groups (2.0% versus 1.8%; *P *= 1.0), or in the incidence of cardiac arrest, tachyarrhythmias, or bradyarrhythmias. Vasopressin and norepinephrine are both potent vasoconstrictors, and animal studies suggest that vasopressin is associated with coronary vasoconstriction and myocardial depression [4,5]. Therefore, we conducted a prospective study to examine, in more detail, ischemic ECG changes, troponin and creatine kinase (CK) levels, and clinical outcomes in a subset of patients enrolled in the VASST Trial. Our specific objectives were to describe troponin and CK levels and ECG abnormalities in patients with severe septic shock and to compare the effect of vasopressin versus that of norepinephrine on troponin and CK levels, ECGs, and the clinical diagnosis of acute myocardial infarction.

## Materials and methods

### Study design

This study evaluating the incidence of cardiac ischemia was a prospective observational substudy performed as part of the Vasopressin And Septic Shock Trial (VASST), a double-blind randomized controlled trial of vasopressin versus norepinephrine in patients with septic shock [3]. We previously published an article assessing intra- and interrater agreement of electrocardiogram interpretation in these patients [6]. VASST enrollment occurred between July 2001 and April 2006. This investigator-initiated substudy started after enrollment in the VASST Trial had begun, and thus was not included in the trial registration (ISRCTN94845869). Nine Canadian sites participated in this substudy, after obtaining local institutional research ethics board approval (St. Paul's Hospital, Vancouver General Hospital, Richmond General Hospital, University Health Network - Toronto General Hospital, University Health Network - Toronto Western Hospital, Mount Sinai Hospital, St. Michael's Hospital, St. Joseph's Hospital, Hotel Dieu Grace Hospital; see Additional file for locations). Written informed consent was obtained from all patients or their surrogates.

### Patients

All patients enrolled in VASST in nine participating centers were included in this substudy. Patients were older than 16 years and had septic shock, defined by the presence of two or more of the systemic inflammatory response syndrome (SIRS) criteria, proven or suspected infection, new dysfunction of at least one organ, and hypotension, despite adequate fluid resuscitation requiring vasopressor support of at least 5 μg/min of norepinephrine (or equivalent) for 6 hours. More-severe septic shock was defined as treatment with 15 μg or more of norepinephrine or the equivalent per minute. Specific cardiac exclusion criteria of VASST were: unstable coronary syndrome (acute myocardial infarction during this episode of shock, based on the combination of history, ECG, and enzyme changes, as defined by the investigator), and underlying chronic heart disease (NYHA class III or IV). Patients were randomized to receive a blinded infusion of study drug, either vasopressin (0.01 to 0.03 U/min) or norepinephrine (5 to 15 µg/min). The study drug and all other vasopressors were titrated and weaned according to protocols, with an initial target mean arterial pressure of 65 to 75 mm Hg. Study-drug infusions were stopped if a serious adverse event was thought to be directly related to study-drug infusion.

### Twelve-lead ECG recording and interpretation

The 12-lead ECGs were recorded at baseline (before study-drug infusion), and 6 hours, 2 days, and 4 days after initiation of the study drug, yielding a total of 373 ECGs. ECGs were read by two reviewers who were blinded to randomization group and troponin levels, by using a checklist to standardize the interpretation. Before the ECG interpretation, a calibration exercise was performed by the two reviewers to refine definitions and maximize interobserver agreement: this exercise and the checklist were previously published [4]. Each ECG was analyzed for rhythm and the presence of Q waves, ST elevation, ST depression, and T-wave inversion. Additionally, the readers assessed whether the ECG was normal or abnormal, and whether, in their opinion, the ECG changes represented ischemia.

### Serum cardiac markers

Troponin I or T, CK, and CK-MB levels were measured in all patients at the same time points as the ECG recordings. Of the nine sites, six measured troponin I (Abbott Laboratories, Abbott Park, IL; or Dade Behring Inc, Newark, DE, USA), two measured troponin T (Roche Diagnostics, Basel, Switzerland), and one switched the assay from troponin I to T during the trial. Table 1 illustrates multiinstitutional laboratory troponin criteria used to diagnose myocardial ischemia or infarction; we also categorized troponin as normal, weakly positive, and highly positive. We further categorized patients regarding troponin elevation. They were categorized as highly positive if they had a highly positive troponin at any time. If they never had a highly positive troponin level, and had a weakly positive troponin level at any time, they were categorized as weakly positive. They were categorized as normal if they had no troponin elevation at any time.

**Table 1:** Troponin I and T interpretation

	Troponin I (µg/L)	Troponin T (µg/L)
Normal	≤0.5, No myocardial injury	<0.05, normal

Weakly positive	0.6 to 2.3, suggestive of myocardial injury	0.05 to 0.09, borderline 0.10 to 0.49, weakly positive

Highly positive	>2.3, suggestive of acute myocardial infarction	>0.49, positive

The two different troponin biomarkers and how their levels were interpreted.

### Ischemia based on ECG

Patients were categorized as having no ischemia, possible ischemia, or probable ischemia, based on ECG interpretations. Patients categorized as having no ischemia were those for whom both readers agreed on the absence of ischemic changes on all of their ECGs. Patients categorized as having probable ischemia were those for whom both readers agreed on the presence of ischemic changes on any single ECG. The remainder of patients were categorized as having possible ischemia, defined as the presence of ischemia on any ECG, without agreement between the two readers. In addition, at each site, using all available clinical data, including troponin levels, the local investigator independently assessed in real time whether the patient met criteria for acute myocardial infarction.

### Statistical analysis

Continuous data are reported as mean and SD, or median and interquartile range (IQR) when not normally distributed. Binary data are reported as counts and proportions. Categoric variables were compared by using the Fisher Exact test, and continuous variables were compared by using the Wilcoxon rank-sum test. Linear and logistic mixed-effects models were used to assess the differences between treatment groups across times, assuming no time trends were present. In logistic regression, we examined factors associated with 28-day mortality in univariate and then in multivariate analysis. Candidate-independent variables were patient age, APACHE II score, more severe shock (defined as treatment with ≥15 μg/min of norepinephrine or the equivalent), history of diabetes, history of ischemic heart disease, vasopressin versus norepinephrine infusion, possible or probable ECG evidence of ischemia, and troponin elevation. Calculations were performed by using R Version 2.8.1 (R Foundation for Statistical Computing, Vienna, Austria).

## Results

Compared with 657 VASST patients who were not enrolled in this substudy, the 121 patients included in this substudy were similar, other than having a higher percentage of males (70.3% versus 59.3%; *P *= 0.026) and higher APACHE II (mean, 28.6 versus 26.8; *P *= 0.007). Of the 121 patients, 65 were randomized to vasopressin, and 56 to norepinephrine; baseline characteristics and outcomes of the two groups were similar (Table 2). Enrolled patients were severely ill, as indicated by APACHE II scores, the proportion with new organ dysfunction, serum lactate levels, and by norepinephrine-infusion doses at study entry.

**Table 2:** Characteristics of patients in the troponin substudy

Variable	Vasopressin (*n *= 65)	Norepinephrine (*n *= 56)
Age, years	62.9 (51.2, 73.6)	65.5 (50.8, 76.1)

Male sex, *n *(%)	43 (66)	42 (75)

Surgical patient, *n *(%)	25 (39)	25 (45)

APACHE II	28.1 (8.0)	29.2 (7.3)

Preexisting conditions, *n *(%) Ischemic heart disease Chronic obstructive pulmonary disease Chronic renal failure Diabetes Liver disease Cancer Solid-organ transplant Corticosteroid use Recent trauma	9 (14) 6 (9) 8 (12) 13 (20) 12 (18) 17 (26) 4 (6) 9 (14) 1 (2)	8 (14) 11 (20) 9 (16) 13 (23) 9 (16) 13 (23) 5 (9) 8 (14) 3 (5)

New organ failure at randomization, *n *(%) Cardiovascular Respiratory Renal Hematology/coagulation Neurologic	65 (100) 58 (89) 43 (66) 20 (31) 21 (32)	56 (100) 50 (89) 41 (73) 13 (23) 18 (32)

Number of organ dysfunctions at randomization	3.0 (2.0, 3.0)	3.0 (2.0, 4.0)

Source of infection, *n *(%) Lung Abdomen Other^a^	28 (43) 19 (29) 18 (28)	28 (50) 16 (29) 12 (21)

Hemodynamic variables at randomization Systolic blood pressure (mm Hg) Mean arterial pressure (mm Hg) Arterial pH Serum lactate (m*M*)	110.0 (98.0, 118.0) 72.0 (67.0, 77.0) 7.34 (7.3, 7.4) 2.3 (1.5, 3.6)	105.0 (95.0, 114.0) 70.0 (65.0, 75.0) 7.36 (7.3, 7.4) 2.2 (1.6, 4.1)

Vasoactive drug dosages at randomization Norepinephrine (μg/min) Epinephrine (μg/min) Dopamine (μg/kg/min) Dobutamine (μg/kg/min) Phenylephrine (μg/min)	14.9 (9.1, 26.6) 30.0 (30.0, 30.0) 3.1 (3.0, 6.0) 6.3 (2.5, 12.5) 66.5 (31.1, 133.2)	18.1 (10.6, 30.0) 25.2 (8.4, 42.0) 8.2 (4.0, 17.0) 7.5 (2.5, 10.0) 104.7 (43.0, 134.5)

Days alive free of mechanical ventilation (first 28 days)	0.5 (0.0, 17.0)	1.0 (0.0, 16.0)

ICU stay,^b ^days	11.5 (6.0, 26.0)	13.0 (6.0, 12.0)

Hospital stay,^b ^days	26.5 (10.5, 74.0)	37.5 (18.5, 89.5)

28-day mortality,^c ^*n *(%)	27 (42)	24 (43)

90-day mortality,^c ^*n *(%)	31 (48)	32 (57)

Baseline characteristics and outcomes of patients randomized to vasopressin and norepinephrine. Continuous variables are presented as median and interquartile range. APACHE II is expressed as mean and standard deviation. APACHE II, Acute Physiology and Chronic Health Evaluation II; ICU, intensive care unit. ^a^Other sites of infection included blood, skin, central nervous system, bones and joints, cardiac system, and reproductive organs. ^b^Censored at day 90 if patient did not die or was not discharged prior to day 90. ICU and hospital stay are from time of admission to the ICU. ^c^Outcome of one patient in the vasopressin group was unknown and excluded from the calculations.

Mean arterial blood pressure (MAP) was similar in the two treatment groups throughout the study, whereas the heart rate was significantly lower in the vasopressin group than in the norepinephrine group during the first 4 days of treatment (*P *= 0.033) (see Figure 1, Additional file). The rate of norepinephrine infusion was significantly lower in the vasopressin group than in the norepinephrine group during the first 4 days (*P *= 0.004) (see Figure 2, Additional file). No statistically significant differences were found in serum creatinine between the norepinephrine and vasopressin groups (Figure 3, Additional file).

### Troponin, CK, and CK-MB levels

Troponin elevation was common, occurring in 26%, 36%, 32%, and 21% of patients at baseline, 6 hours, day 2, and day 4, respectively. No differences were seen in baseline characteristics, organ failures, or mortality between patients with normal, weakly positive, or highly positive troponin levels, except that patients with weakly or highly positive troponin had a higher incidence of underlying ischemic heart disease (Table 3, and Table 7, Additional file). In addition, the rates of acute myocardial infarction, diagnosed by local site investigators, were higher in the patients with elevated troponin (Table 3).

**Table 3:** Baseline characteristics and outcomes of patients with and without elevations in serum troponin

	Troponin levels (*N *= 120)^a^			
	
Variable	Normal (*n *= 72)	Weakly positive^b^ (*n *= 30)	Highly positive^b^ (*n *= 18)	*P *value
Age, years	62.7 (51.1, 72.6)	68.4 (47.8, 77.8)	63.2 (50.0, 73.4)	0.378

Male, *n *(%)	51 (71)	22 (73)	12 (67)	0.920

Surgical patient, *n *(%)	34 (47)	8 (27)	7 (39)	0.152

APACHE II	27.5 (7.6)	29.7 (6.6)	31.7 (8.6)	0.153

Preexisting conditions, *n *(%)				
Ischemic heart disease	6 (8)	8 (27)	3 (17)	0.041
Diabetes	14 (19)	9 (30)	3 (17)	0.469
Corticosteroid use	8 (11)	7 (23)	2 (11)	0.269

New organ failure at randomization, *n *(%)				
Respiratory	66 (92)	25 (83)	16 (89)	0.406
Renal	49 (68)	21 (70)	14 (78)	0.781
Hematology/coagulation	17 (24)	8 (27)	7 (39)	0.428
Neurologic	20 (28)	14 (47)	5 (28)	0.173

Clinical MI,^c ^*n *(%)	0 (0)	2 (7)	3 (17)	0.004

28-day mortality,^d ^*n *(%)	30 (42)	12 (41)	8 (44)	1.0

90-day mortality,^d ^*n *(%)	40 (56)	14 (48)	8 (44)	0.612

Among 120 patients, the baseline characteristics and outcomes of patients with or without elevated serum troponin. Age is presented as median and first and third quartiles. APACHE II is presented as mean and standard deviation. *P *value is based on the Fisher Exact test or Wilcoxon rank-sum test. ^a^One patient had no troponin data. ^b^See Table 1 for definitions of weakly positive and highly positive troponin levels. Patients were categorized based on ever having highly positive or weakly positive troponin. ^c^MI, myocardial infarction. At each site, the local investigator independently assessed in real time whether the patient met clinical criteria for myocardial infarction. ^d^Outcome of one patient in the vasopressin group was unknown and excluded from the calculation.

Five patients (two in the vasopressin group, three in the norepinephrine group) had acute myocardial infarction diagnosed clinically, using symptoms, ECGs, and serum cardiac biomarkers. No significant differences were seen in troponin, CK, and CK-MB levels in patients randomized to vasopressin versus norepinephrine at any of the times (Table 4). Further, the percentages of patients with weakly positive troponin (27.6% versus 22.7%) and highly positive troponin (13.8% versus 15.2%) were similar between the vasopressin and norepinephrine groups, respectively.

**Table 4:** Troponin, CK, and CK-MB levels at each time point in patients randomized to vasopressin or norepinephrine

	Vasopressin group median (1^st^, 3^rd ^quartile), *n*				Norepinephrine group median (1^st^, 3^rd ^quartile), *n*				
		
Time	Baseline	6 hours	Day 2	Day 4	Baseline	6 hours	Day 2	Day 4	*P *value
Troponin I (µg/L)	0.07 (0.0, 0.54) 48	0.11 (0.0, 0.71) 44	0.15 (0.0, 0.60) 39	0.03 (0.0, 0.24) 35	0.20 (0.0, 0.39) 44	0.35 (0.10,0.70) 39	0.21 (0.0, 0.60) 37	0.14 (0.0, 0.43) 33	0.230

Troponin T (µg/L)	0.06 (0.02, 0.09) 14	0.06 (0.0, 0.14) 11	0.03 (0.0, 0.09) 12	0.00 (0.0, 0.02) 9	0.01 (0.0, 0.42) 11	0.06 (0.0, 0.38) 10	0.07 (0.01, 0.35) 12	0.13 (0.01, 0.39) 8	0.360

CK (U/L)	204 (61, 487) 54	208 (54, 435) 48	190 (36, 452) 50	69 (21, 189) 45	184 (60, 309) 46	192 (68, 438) 46	147 (41, 296) 45	46 (21, 222) 39	0.985

CK-MB (µg/L)	4.0 (1.6, 6.9) 16	3.0 (1.3, 6.0) 15	5.0 (1.0, 16.0) 14	1.4 (0.0, 3.1) 14	4.3 (1.0, 8.0) 14	2.6 (1.8, 17.2) 12	4.7 (2.0, 16.0) 13	1.9 (1.0, 6.0) 10	0.396

Serum levels of troponin, creatine kinase (CK), and creatine kinase-MB (CK-MB) at each time point. *P *value is based on linear mixed-effects regression model and is for the null hypothesis of no difference in troponin/CK/CK-MB level between the treatment groups at 6 hours, day 2, and day 4, adjusted for baseline value. Outcome variables were log transformed if they were not normally distributed.

### Electrocardiograms and ischemia

Table 5 presents blinded interpretation of ECGs in patients randomized to vasopressin versus norepinephrine. More patients in the norepinephrine than in the vasopressin group had Q waves (baseline, 6 hours, day 2, and day 4); no other differences appeared in ECGs between groups. In the pooled cohort, no association was present between specific ECG findings or the presence of ECG ischemia at any time, and 28-day mortality (Table 8, Additional file).

**Table 5:** ECG interpretation at each time point in patients randomized to vasopressin or norepinephrine (*N *= 121)

	Vasopressin (*n *= 65)				Norepinephrine (*n *= 56)				*P *value
		
Variable, *n *(%)	Baseline (*n *= 61)	6 hours (*n *= 43)	Day 2 (*n *= 44)	Day 4 (*n *= 39)	Baseline (*n *= 54)	6 hours (*n *= 40)	Day 2 (*n *= 48)	Day 4 (*n *= 35)	
Normal^a^	19 (31)	15 (35)	14 (32)	19 (49)	10 (19)	12 (30)	14 (29)	8 (23)	0.267

Ischemia^a^	9 (15)	6 (14)	8 (18)	3 (8)	9 (17)	2 (5)	3 (6)	3 (9)	0.099

Atrial fib/ flutter/PSVT	6 (10)	10 (23)	7 (16)	4 (10)	15 (28)	13 (33)	14 (29)	8 (23)	0.427

Bundle branch block	6 (10)	4 (10)	6 (14)	4 (10)	7 (13)	4 (10)	7 (15)	7 (20)	0.359

ST elevation	6 (10)	4 (10)	3 (7)	2 (5)	4 (7)	3 (8)	3 (6)	1 (3)	0.869

ST depression	5 (9)	4 (10)	6 (14)	1 (3)	8 (15)	2 (5)	2 (4)	3 (9)	0.317

T inversion	10 (16)	10 (23)	8 (18)	6 (15)	12 (22)	8 (20)	11 (23)	8 (23)	0.530

Q wave	11 (18)	3 (7)	6 (14)	7 (18)	12 (22)	8 (20)	10 (21)	9 (26)	0.039

Ventricular rhythm	0 (0)	0 (0)	0 (0)	0 (0)	1 (2)	0 (0)	1 (2)	0 (0)	-^b^

Arrhythmia^c^	6 (10)	10 (23)	7 (16)	4 (10)	16 (30)	13 (33)	15 (31)	8 (23)	0.339

ECG findings at each time point for patients randomized to vasopressin and norepinephrine. Values are expressed as *n *(%). *P *value is based on logistic mixed-effects model and is for the null hypothesis of no difference in ECG interpretation between the treatment groups at 6 hours, day 2, and day 4, adjusted for baseline interpretation. ECG interpretation results are based only on the data of Reader 2, blinded to randomization group and troponin level. ^a^The reader was asked for an overall assessment of whether the ECG was normal or abnormal, and whether, in his opinion, the ECG changes represented ischemia. ^b^Event rate is too low for *P*-value calculation. ^c^Arrhythmia includes atrial fibrillation, atrial flutter, paroxysmal supraventricular tachyarrhythmia, and ventricular rhythm.

We were interested to determine whether the presence or absence of cardiac ischemia on ECG was associated with different clinical outcomes. No differences were found in baseline characteristics or outcomes in patients with (*n *= 56) or without (*n *= 60) ECG ischemia (Table 6), even if troponin was elevated (Table 9, Additional file). Sixty (51.7%) patients had no ischemia on any ECG at any time; 39 (33.6%) patients had possible ECG ischemia (defined as ischemia on any one ECG without agreement between the two readers), and 17 (14.6%) patients had probable ECG ischemia (defined as ischemia on any one ECG, and both readers agreed).

**Table 6:** Comparison of patients with ischemia versus no ischemia on ECG.

Variable	Ischemia (N =56)	No ischemia^a ^(N = 60)	*P *value
Age, years	64.1 (48.0, 77.1)	63.0 (53.5, 73.3)	0.808

Male, *n *(%)	40 (71)	41 (68)	0.840

Surgical patient, *n *(%)	21 (38)	25 (42)	0.706

APACHE II	29.9 (7.5)	27.4 (8.0)	0.150

Preexisting conditions, *n *(%)			
Ischemic heart disease	10 (18)	4 (7)	0.088
Diabetes	13 (23)	10 (17)	0.486

Baseline lactate	2.0 (1.6, 4.5)	2.6 (1.5, 3.9)	0.441

More-severe shock,^b ^*n *(%)	28 (50)	33 (55)	0.710

Vasopressin, *n *(%)	31 (55)	32 (53)	0.854

Days alive free of mechanical ventilation (first 28 days)	0.0 (0.0, 13.0)	2.0 (0.0, 17.0)	0.165

ICU stay,^c ^days	13.0 (6.0, 31.0)	11.5 (6.0, 20.0)	0.518

Hospital stay,^c ^days	27.0 (11.0, 77.0)	35.5 (16.0, 77.5)	0.703

28-day mortality,^d ^*n *(%) 95% CI	26 (47) 34%-60%	22 (37) 24%-49%	0.263

90-day mortality,^d ^*n *(%) 95% CI	33 (60) 47% to 73%	27 (45) 32% to 58%	0.136

Baseline and outcome data for patients with ischemia and those with no ischemia on ECG.

Continuous variables are presented as median and first and third quartiles. Categoric variables are presented as number and percentage. *P *value is based on Fisher Exact test or Wilcoxon rank-sum test. Five patients had no data for ECG diagnosis of ischemia. ^a^Defined as no ischemia on any ECG for both readers. ^b^Defined as treatment with 15 μg/min of norepinephrine or the equivalent at randomization. ^c^ICU and hospital stay from time of admission to ICU. ^d^Mortality outcome of one patient was unknown and excluded from the calculation.

### Logistic regression

In the univariable analysis, only age (OR, 1.03; 95% CI, 1.00 to 1.05; *P *= 0.028), APACHE II (OR, 1.08; 95% CI, 1.02 to 1.14; *P *= 0.005) and the presence of more-severe shock (OR, 2.32; 95% CI, 1.11 to 4.88; *P *= 0.025) were associated with 28-day mortality. In the multivariable analysis, only APACHE II score (OR, 1.07; 95% CI, 1.01 to 1.14; *P *= 0.033) was highly associated with 28-day mortality.

### Sensitivity analyses

ECGs and troponin assays were not performed for some patients at some of the time points. Such missing data may result in an overestimate of abnormalities we observed, given that patients for whom clinicians recorded ECGs and troponin levels have a greater likelihood of being labeled abnormal. We thus repeated our analyses in two different ways. First, we used only baseline ECG reading and troponin level as predictors. In a second analysis, we used the ECG reading and troponin level as time-dependent covariates. Our overall results did not change in either analysis.

## Discussion

Regarding our first objective, in this prospective substudy of patients who had septic shock enrolled in VASST, troponin elevations were very common, occurring in up to 36% of patients. However, troponin elevation in patients with septic shock was not associated with increased mortality, compared with patients with no troponin elevation. Cardiac ischemia on ECGs was also common, occurring in 48% of patients at one or more of the measured time points; however, cardiac ischemia on ECG was not predictive of worse outcomes.

Regarding our second objective, no significant differences were found in troponin and creatine kinase levels between vasopressin- and norepinephrine-treated patients. In addition, the overall incidence of ECG cardiac ischemia was similar in the vasopressin- and norepinephrine-treated patients, although more patients in the norepinephrine group had Q waves. No difference was noted between vasopressin and norepinephrine groups in the clinical diagnosis of acute myocardial infarction.

High doses of norepinephrine may cause myocardial damage, and this in turn may increase troponin levels; nonetheless, despite lower norepinephrine doses in the vasopressin group than in the norepinephrine group, no overall difference was noted in markers of myocardial ischemia such as troponin, creatine kinase levels, and ECG findings. It is relevant to note that heart rate was significantly lower in the vasopressin group than in the norepinephrine group over the first 4-day period (*P *= 0.033), yet again, we found no overall difference in markers of myocardial ischemia. We did not find evidence that renal dysfunction contributed to any differences between vasopressin and norepinephrine groups in markers of myocardial ischemia, in that our marker of renal function (serum creatinine over time) was not different between groups. These observations highlight the complexity and multiple factors that could contribute to myocardial ischemia in patients who have septic shock.

In critically ill patients, many mechanisms are likely responsible for the release of troponin into the systemic circulation, including impaired microvascular perfusion, reduced oxygen delivery to the heart, myocardial depression and cellular injury, and increased myocardial oxygen demand [7]. Although cardiac troponins are sensitive and relatively specific biomarkers of myocardial injury, elevations are not diagnostically specific for acute coronary syndrome, and can occur in the absence of coronary atherosclerotic disease [8]. Cardiac troponin elevation may be even less specific in critically ill patients, as many conditions are associated with increased troponin, including infection, sepsis, septic shock, hypotension, arrhythmias, pulmonary embolism, and renal insufficiency [9]. Troponin levels are frequently elevated in critically ill patients and are independently positively associated with short- and long-term mortality [10-13]. In a systematic review, 43% of 3278 critically ill patients enrolled in 20 studies had elevated troponin levels [12]. In adjusted analyses, elevated troponin was associated with an increased risk of death (OR, 2.5; 95% CI, 1.9 to 3.4; *P *< 0.001); in unadjusted analyses, it was associated with increased length of ICU stay of 3 days (95% CI, 1.0 to 5.1 days; *P *= 0.004) and hospital stay of 2.2 days (95% CI, -0.6 to 4.9 days; *P *= 0.12).

Although we found a similarly high prevalence of elevated troponin (approximately 30% to 35% of patients) in our study of patients who had septic shock, we did not find that increased troponin was associated with increased mortality. Published data regarding the prognostic value of troponin elevation in patients with sepsis syndrome are conflicting [14-22]. However, consistent with our results, most studies have not found that troponin elevation is an independent predictor of mortality, after adjusting for other variables [14,16-18]. In multivariate analyses, previous trials have identified greater severity of illness and the presence of shock as independent predictors of death, as we did in our study [14,16-18]. In contrast, whereas several small studies found increased mortality in patients with sepsis who have elevated troponin levels [19-21], only one study concluded that elevated cTnI was an independent prognosticator of mortality after adjusting for other significant variables (OR, 2.020; 95% CI, 1.153 to 3.541; *P *= 0.014) [22]. It is not clear whether elevated troponin is not prognostic in septic shock, or whether patient heterogeneity, or differences in timing and frequency of troponin measurement in different studies, accounts for these conflicting results.

ECGs are used in addition to serum cardiac biomarkers to diagnose cardiac ischemia in critically ill patients who are unable to communicate symptoms. However, ECG findings suggestive of ischemia, such as ST-segment deviations, are common in critically ill patients and may be nonspecific for diagnosis of acute cardiac ischemia. In our study, 48% of patients with septic shock had possible or probable ECG ischemia; however, this was not prognostic of worse clinical outcomes, even with concomitant troponin elevation. Our prevalence rate of 48% is generally consistent with that of other relevant studies. Landesberg and colleagues [23] monitored continuous 12-lead ECGs in 101 general ICU patients with known coronary artery disease, or two or more risk factors for coronary artery disease [23]. Of these patients, 21% had ischemic ST-segment changes, characterized in most by ST depression. Similarly, Kress and colleagues [24] observed ST-segment elevation or depression on continuous three-lead Holter monitoring in 24% of critically ill patients with risk factors for coronary artery disease, which was associated with a longer ICU stay. In a prospective trial evaluating myocardial ischemia during mechanical ventilation and weaning, Frazier and colleagues [25] observed ST-segment changes in 70% of 43 patients, and these patients were 60% more likely to fail an initial weaning trial.

Both vasopressin and norepinephrine are potent vasoconstrictors. In VASST, no differences were found in cardiac (clinical ischemia or acute myocardial infarction based on symptoms, ECGs, and biomarkers), mesenteric, cerebrovascular, or digital ischemic events in the vasopressin- and norepinephrine-treated groups [3]. Similarly, in this VASST substudy, the rates of ECG ischemia and troponin elevations were similar in the vasopressin and norepinephrine groups. We did find a significantly higher rate of Q waves in the norepinephrine group compared with the vasopressin group. Several small uncontrolled studies [26-29] found an occasional occurrence of cardiac ischemia during infusion of vasopressin, but the small sample size and lack of controls preclude conclusions regarding risk of cardiac ischemia due to vasopressin infusion. In contrast, vasopressin infusion is cardioprotective in models of myocardial ischemia [30,31] and in patients with postcardiotomy shock [32]. In other studies, norepinephrine infusion was associated with cardiac ischemia and elevation of serum troponin. In the study by Landesberg and colleagues [23], norepinephrine infusion significantly predicted prolonged (>60 minutes) ischemia (OR, 3.08; CI, 0.99 to 9.60; *P *= 0.05) and troponin elevation in 101 general ICU patients. To the extent that vasopressin versus norepinephrine have different effects on overall cardiac function, we also found no difference between the effects of vasopressin and norepinephrine on cardiac output and cardiac function in another substudy of VASST [33].

The strengths of this substudy are the inclusion of patients meeting stringent criteria for septic shock, the multicenter representation, and prospective collection of both ECGs and troponin at four time points. Previous studies describing troponin levels in patients with sepsis did not evaluate concurrent ECGs. The ECG readers were blinded to randomization group and cardiac biomarkers. Concealed randomization of patients to blinded vasopressin or norepinephrine infusions permitted an unbiased comparison between the two groups.

This study has limitations. Although our sample size is modest, it is similar to or larger than that of many previous studies evaluating troponin levels in patients with sepsis [15-17,19,20]; however, we cannot exclude the possibility that our study was underpowered to detect differences in important variables. Given that patients in the vasopressin group were also receiving norepinephrine, this study is not a direct comparison of the administration of vasopressin and norepinephrine. Our study was conducted before the introduction of sensitive troponin assays, with the potential that the older-generation troponin assays were affected by renal insufficiency, resulting in false-positive troponin elevation. However, this would apply to both groups of patients, as serum creatinine was similar in the vasopressin- and norepinephrine-treated patients. The generalizability of our results is limited by our exclusion of patients with unstable coronary syndrome (acute myocardial infarction based on the combination of history, ECG, and enzyme changes),underlying chronic heart disease (NYHA III and IV), anticipated to die within 12 hours, or receiving open-label vasopressin. Given these exclusions, our study population may have been at low to moderate risk of coronary ischemia, and would thus underestimate the true prevalence of ischemia in a less restricted septic shock population. The lack of a reference standard for ECG interpretation precludes the accurate diagnosis of ischemia.

Finally, we do not have data on cardiac output, central venous saturation, or cardiac ultrasound; thus, we cannot comment on whether the observed troponin elevations indicate subclinical or more overt cardiac ischemia and dysfunction.

## Conclusions

Troponin elevation and ECG evidence of ischemia were common, occurring in 36% and 48%, respectively, of a subgroup of patients with septic shock enrolled in VASST. No significant differences in serum troponin levels and ECG evidence of ischemia were found between the vasopressin and norepinephrine groups. In this study, neither increased troponin nor ECG evidence of ischemia predicted increased mortality risk.

## Key messages

• In this prospective substudy of patients with septic shock enrolled in VASST, troponin elevations were very common, occurring in up to 36% of patients.

• Troponin elevation in patients with septic shock was not associated with increased mortality, compared with patients with no troponin elevation.

• Cardiac ischemia on ECGs was also common, occurring in 48% of patients at one or more of the measured times; however, cardiac ischemia on ECG was not predictive of worse outcomes.

• No significant differences in troponin and creatine kinase levels were found between patients treated with vasopressin and with norepinephrine, compared with those treated with norepinephrine alone. In addition, the overall incidence of ECG cardiac ischemia was similar in the two groups, and no difference was noted in the clinical diagnosis of acute myocardial infarction.

## Abbreviations

APACHE II: Acute Physiology and Chronic Health Evaluation II; CK: creatine kinase; ECG: electrocardiogram; ICU: intensive care unit; IQR: interquartile range; NE: norepinephrine; NYHA: New York Heart Association; OR: odds ratio; SD: standard deviation; SIRS: systemic inflammatory response syndrome; VASST: Vasopressin in Severe Sepsis Trial (*N Engl J Med *2008; 358:877-887); VP: vasopressin.

## Competing interests

The authors declare that they have no competing interests.

## Authors' contributions

SM participated in conception and design, drafting of the article, collection and assembly of data, administrative, technical, or logistic support, and final approval of the article; JG, conception and design, collection and assembly of data, critical revision of the article for important intellectual content, and final approval of the article; ACG, collection and assembly of data, critical revision of the article for important intellectual content, and final approval of the article; DJCook, statistical expertise, critical revision of the article for important intellectual content, and final approval of the article; SL and GN, collection and assembly of data, final approval of the article; KB, AL, and CS, collection and assembly of data, administrative, technical, or logistic support, final approval of the article; DA, JS, and TCKL, collection and assembly of data, statistical expertise, critical revision of the article for important intellectual content, final approval of the article; KRW, collection and assembly of data, critical revision of the article for important intellectual content, final approval of the article; MS, collection and assembly of data, administrative, technical, or logistic support, and final approval of the article; DJCooper, collection and assembly of data; critical revision of the article for important intellectual content, and final approval of the article; CLH, collection and assembly of data, critical revision of the article for important intellectual content, final approval of the article; PH, collection and assembly of data, critical revision of the article for important intellectual content, final approval of the article; JP, collection and assembly of data, critical revision of the article for important intellectual content, final approval of the article; and JAR, conception and design, collection and assembly of data, statistical expertise, critical revision of the article for important intellectual content, and final approval of the article.
