# Erratum to: Cardiac ischemia in patients with septic shock randomized to vasopressin or norepinephrine

**DOI:** 10.1186/s13054-017-1680-7

**Published:** 2017-05-04

**Authors:** Sangeeta Mehta, John Granton, Anthony C. Gordon, Deborah J. Cook, Stephen Lapinsky, Gary Newton, Kris Bandayrel, Anjuli Little, Chuin Siau, Dieter Ayers, Joel Singer, Terry C. K. Lee, Keith R. Walley, Michelle Storms, D. James Cooper, Cheryl L. Holmes, Paul Hebert, Jeffrey Presneill, James A. Russell, for the Vasopressin and Septic Shock Trial (VASST) Investigators

**Affiliations:** 1 Department of Medicine and Interdepartmental Division of Critical Care Medicine, Mount Sinai Hospital, University of Toronto, Toronto, Ontario Canada; 2 University Health Network-General Division, University of Toronto, Toronto, Ontario Canada; 3 Intensive Care, Charing Cross Hospital, Imperial, College, London, UK; 4 Departments of Medicine, Clinical Epidemiology & Biostatistics, McMaster University, Hamilton, Ontario Canada; 5 Department of Medicine and Division of Cardiology, Mount Sinai Hospital, University of Toronto, Toronto, Ontario, Canada; 6 Department of Respiratory Medicine, Changi General Hospital, Singapore, Singapore; 7 School of Population and Public Health, Centre for Health Evaluation and Outcome Sciences, and University of British Columbia, Vancouver, British Columbia Canada; 8 Critical Care Research, Laboratories, Heart and Lung Institute, St. Paul’s Hospital, and University of British Columbia, Vancouver, British Columbia Canada; 9 Alfred Hospital, Monash University, Melbourne, Australia; 10 Kelowna General Hospital, Kelowna, and University of British Columbia, Kelowna, British Columbia Canada; 11 Ottawa Hospital-General Campus, and University of Ottawa, Ottawa, Ontario Canada; 12 Mater Health Services, University of Queensland and Monash University, South Brisbane, Queensland Australia

## Erratum

Unfortunately this article [1] published without its additional files (Additional files 1, 2, 3, 4, 5, 6 and 7). Please see these listed below and attached as additional files to this erratum.

## Additional files

Additional file 1: Participating sites. (DOCX 13 kb)

Additional file 2: Table S7. Baseline characteristics and outcomes in vasopressin and norepinephrine-treated patients, grouped by serum troponin levels. (DOCX 17 kb)

Additional file 3: Table S8. Specific ECG diagnoses and patient outcomes. (DOCX 14 kb)

Additional file 4: Table S9. Outcomes related to ECG ischemia for all patients and for patients with troponin elevation. (DOCX 15 kb)

Additional file 5: Figure S1. Comparison of mean arterial pressure and heart rate in the norepinephrine group and vasopressin group. (DOCX 122 kb)

Additional file 6: Figure S2. Rates of total norepinephrine infusion (open-label and study drug) in the vasopressin treated group and the norepinephrine treated group. (DOCX 60 kb)

Additional file 7: Figure S3. Serum creatinine in the vasopressin treated group and the norepinephrine treated group. (DOCX 64 kb)
