# Supplementary material for: Erratum to: Cardiac ischemia in patients with septic shock randomized to vasopressin or norepinephrine
Source: Crit Care. 2017 May 4;21:98. doi: 10.1186/s13054-017-1680-7 (PMC5415714; doi:10.1186/s13054-017-1680-7)
Supplement: Supplementary file 1 — Participating sites. (DOCX 13 kb) [file 13054_2017_1680_MOESM1_ESM.docx]

Additional file 1

Participating Sites

St. Paul’s Hospital, Vancouver, British Columbia

Vancouver General Hospital, Vancouver, British Columbia

Richmond General Hospital, Vancouver, British Columbia

University Health Network - Toronto General Hospital, Toronto, Ontario

University Health Network - Toronto Western Hospital, Toronto, Ontario

Mount Sinai Hospital, Toronto, Ontario

St. Michael's Hospital, Toronto, Ontario

St. Joseph's Hospital, Hamilton, Ontario

Hotel Dieu Grace Hospital, Windsor, Ontario
