# Supplementary material for: Erratum to: Cardiac ischemia in patients with septic shock randomized to vasopressin or norepinephrine
Source: Crit Care. 2017 May 4;21:98. doi: 10.1186/s13054-017-1680-7 (PMC5415714; doi:10.1186/s13054-017-1680-7)
Supplement: Supplementary file 2 — Baseline characteristics and outcomes in vasopressin and norepinephrine-treated patients, grouped by serum troponin levels. (DOCX 17 kb) [file 13054_2017_1680_MOESM2_ESM.docx]

Additional file 2: Table S7. Baseline characteristics and outcomes in vasopressin and norepinephrine-treated patients, grouped by serum troponin levels.

|  | Vasopressin (N=64)  Troponin levels | | | | Norepinephrine (N=56)  Troponin levels | | | | P  value^c^ |
| --- | --- | --- | --- | --- | --- | --- | --- | --- | --- |
| Variable | Normal  (N=41) | Weakly  positive  (N=14) | Highly positive  (N=9) | P  value | Normal  (N=31) | Weakly  positive  (N=16) | Highly  positive  (N=9) | P  value |  |
| Age, years | 61.6  (51.2, 71.7) | 67.9  (61.1, 77.3) | 59.2  (43.6, 79.2) | 0.534 | 63.2  (51.1, 75.3) | 70.8  (47.1, 79.3) | 64.1  (58.6, 68.7) | 0.658 | 0.445 |
| Male | 25 (61) | 10 (71) | 8 (89) | 0.294 | 26 (84) | 12 (75) | 4 (44) | 0.056 | 0.913 |
| Surgery | 18 (44) | 3 (21) | 3 (33) | 0.306 | 16 (52) | 5 (31) | 4 (44) | 0.463 | 0.168 |
| APACHE II | 26.4 (7.6) | 29.6 (7.5) | 34.8 (7.2) | 0.026 | 29.1 (7.6) | 29.8 (5.9) | 28.7 (9.3) | 0.873 | 0.104 |
| Preexisting conditions |  |  |  |  |  |  |  |  |  |
| Ischemic heart disease | 1 (2) | 6 (43) | 2 (22) | <.001 | 5 (16) | 2 (13) | 1 (11) | 1.000 | 0.054 |
| Diabetes | 6 (15) | 4 (29) | 3 (33) | 0.258 | 8 (26) | 5 (31) | 0 (0) | 0.173 | 0.396 |
| Steroid | 5 (12) | 4 (29) | 0 (0) | 0.119 | 3 (10) | 3 (18) | 2 (22) | 0.453 | 0.443 |
| Organ failures |  |  |  |  |  |  |  |  |  |
| Respiratory | 39 (95) | 10 (71) | 8 (89) | 0.036 | 27 (87) | 15 (94) | 8 (89) | 0.850 | 0.436 |
| Renal | 25 (61) | 9 (64) | 9 (100) | 0.071 | 24 (77) | 12 (75) | 5 (56) | 0.455 | 0.736 |
| Hematology/coagulation | 9 (22) | 5 (36) | 5 (56) | 0.123 | 8 (26) | 3 (19) | 2 (22) | 0.909 | 0.399 |
| CNS | 12 (29) | 6 (43) | 3 (33) | 0.684 | 8 (26) | 8 (50) | 2 (22) | 0.204 | 0.126 |
| Clinical MI^a^ | 0 (0) | 2 (14) | 0 (0) | - | 0 (0) | 0 (0) | 3 (33) | - | - |
| 90 day mortality^b^ | 19 (46) | 7 (54) | 4 (44) | 0.932 | 21 (68) | 7 (44) | 4 (44) | 0.194 | 0.566 |

Legend for Table 7. In this table we present baseline characteristics, organ failures, rates of myocardial infarction (as judged by investigators) and number of deaths in norepinephrine and vasopressin-treated patients, categorized by serum troponin levels.

Patients were categorized based on ever having highly positive or weakly positive troponin at any time point.

Age is presented as median and first and third quartiles. APACHE II is presented as mean and standard deviation.

P value within each treatment group is based on Fisher’s exact test or Wilcoxon rank-sum test.

a. Not enough events for P value calculation.

b. Outcome of one patient in the vasopressin group was unknown and excluded from the calculation.

c. P value represents comparison between the three troponin level categories adjusted for treatment group, and is based on linear or logistic regression.
