# Supplementary material for: Erratum to: Cardiac ischemia in patients with septic shock randomized to vasopressin or norepinephrine
Source: Crit Care. 2017 May 4;21:98. doi: 10.1186/s13054-017-1680-7 (PMC5415714; doi:10.1186/s13054-017-1680-7)
Supplement: Supplementary file 3 — Specific ECG diagnoses and patient outcomes. (DOCX 14 kb) [file 13054_2017_1680_MOESM3_ESM.docx]

Additional file 3: Table S8. Specific ECG diagnoses and patient outcomes (N=121)

| ECG interpretation | Patients  N (%) | | 28 day mortality  N (%) | 90 day mortality  N (%) |
| --- | --- | --- | --- | --- |
|  | Reader 1 | Reader 2 |  |  |
| Atrial Fib/flutter/PSVT, N (%) | 44 (36) | 46 (38) | 24 (54) | 29 (64) |
| Bundle branch block, N (%) | 24 (20) | 19 (16) | 9 (47) | 12 (63) |
| ST elevation, N (%) | 11 (9) | 21 (18) | 8 (40) | 11 (55) |
| ST depression, N (%) | 32 (27) | 28 (24) | 12 (44) | 14 (52) |
| T wave inversion, N (%) | 55 (47) | 41 (35) | 14 (35) | 21 (53) |
| Q wave, N (%) | 26 (22) | 38 (32) | 16 (42) | 19 (50) |
| Patients with ischemia on baseline ECG, N (%) | 15 (14) | 18 (17) | 7 (39) | 10 (56) |
| Patients with ≥1 ischemic ECG, N (%) | 38 (32) | 38 (32) | 16 (43) | 20 (54) |
| Patients with no ischemia on any ECG, N (%) | 80 (68) | 79 (68) | 33 (42) | 41 (52) |
| Patients whose ECGs were all normal, N (%) | 29 (24) | 9 (7) | 4 (44) | 4 (44) |

Legend for Table 8. In this table we present ECG findings at any time point. ECG readers were blinded to randomization group and serum troponin levels. Mortality data correspond to Reader 2 results.
