# Supplementary material for: Erratum to: Cardiac ischemia in patients with septic shock randomized to vasopressin or norepinephrine
Source: Crit Care. 2017 May 4;21:98. doi: 10.1186/s13054-017-1680-7 (PMC5415714; doi:10.1186/s13054-017-1680-7)
Supplement: Supplementary file 4 — Outcomes related to ECG ischemia for all patients and for patients with troponin elevation. (DOCX 15 kb) [file 13054_2017_1680_MOESM4_ESM.docx]

Additional file 4: Table S9. Outcomes related to ECG ischemia for all patients and for patients with troponin elevation.

|  | Ischemia^a^ | | |  |
| --- | --- | --- | --- | --- |
| Variable & troponin elevation | No | Possible | Probable | P value |
| N (%) |  |  |  |  |
| Regardless of troponin | 60 | 39 | 17 | - |
| With troponin elevation ^b^ | 19 | 16 | 12 | - |
| Proportion discharged from ICU in 28 days or less |  |  |  |  |
| Regardless of troponin | 0.44 | 0.29 | 0.29 | 0.369 |
| With troponin elevation | 0.32 | 0.38 | 0.25 | 0.633 |
| Proportion discharged from hospital in 28 days or less |  |  |  |  |
| Regardless of troponin | 0.15 | 0.21 | 0.06 | 0.360 |
| With troponin elevation | 0.21 | 0.31 | 0.08 | 0.804 |
| 28 day mortality, N (%) |  |  |  |  |
| Regardless of troponin | 22 (37) | 19 (50) | 7 (41) | 0.401 |
| With troponin elevation | 8 (42) | 5 (33) | 6 (50) | 0.644 |
| 90 day mortality, N (%) |  |  |  |  |
| Regardless of troponin | 27 (45) | 25 (66) | 8 (47) | 0.122 |
| With troponin elevation | 8 (42) | 7 (47) | 6 (50) | 0.932 |

Legend for Table 9. In this table we present outcomes for all patients, and for patients with positive troponin, according to the presence of no, possible, or probable ECG ischemia. No Ischemia is defined as no ischemia on all ECGs for both readers. Possible is defined as ischemia on any 1 ECG, but both readers disagree. Probable is defined as ischemia on any 1 ECG and both readers agree.

Results were based on ECG interpretation blinded to randomization group and troponin levels.

P value is for the null hypothesis of no difference in the variable across the ischemia outcome based on Fisher’s exact test or Gray’s test for cumulative incidence functions.

a. Data for ECG diagnosis for ischemia is missing for 5 patients.

b. Includes patients with weakly positive and highly positive troponins.
