# Supplementary material for: Erratum to: Cardiac ischemia in patients with septic shock randomized to vasopressin or norepinephrine
Source: Crit Care. 2017 May 4;21:98. doi: 10.1186/s13054-017-1680-7 (PMC5415714; doi:10.1186/s13054-017-1680-7)
Supplement: Supplementary file 5 — Comparison of mean arterial pressure and heart rate in the norepinephrine group and vasopressin group. (DOCX 122 kb) [file 13054_2017_1680_MOESM5_ESM.docx]

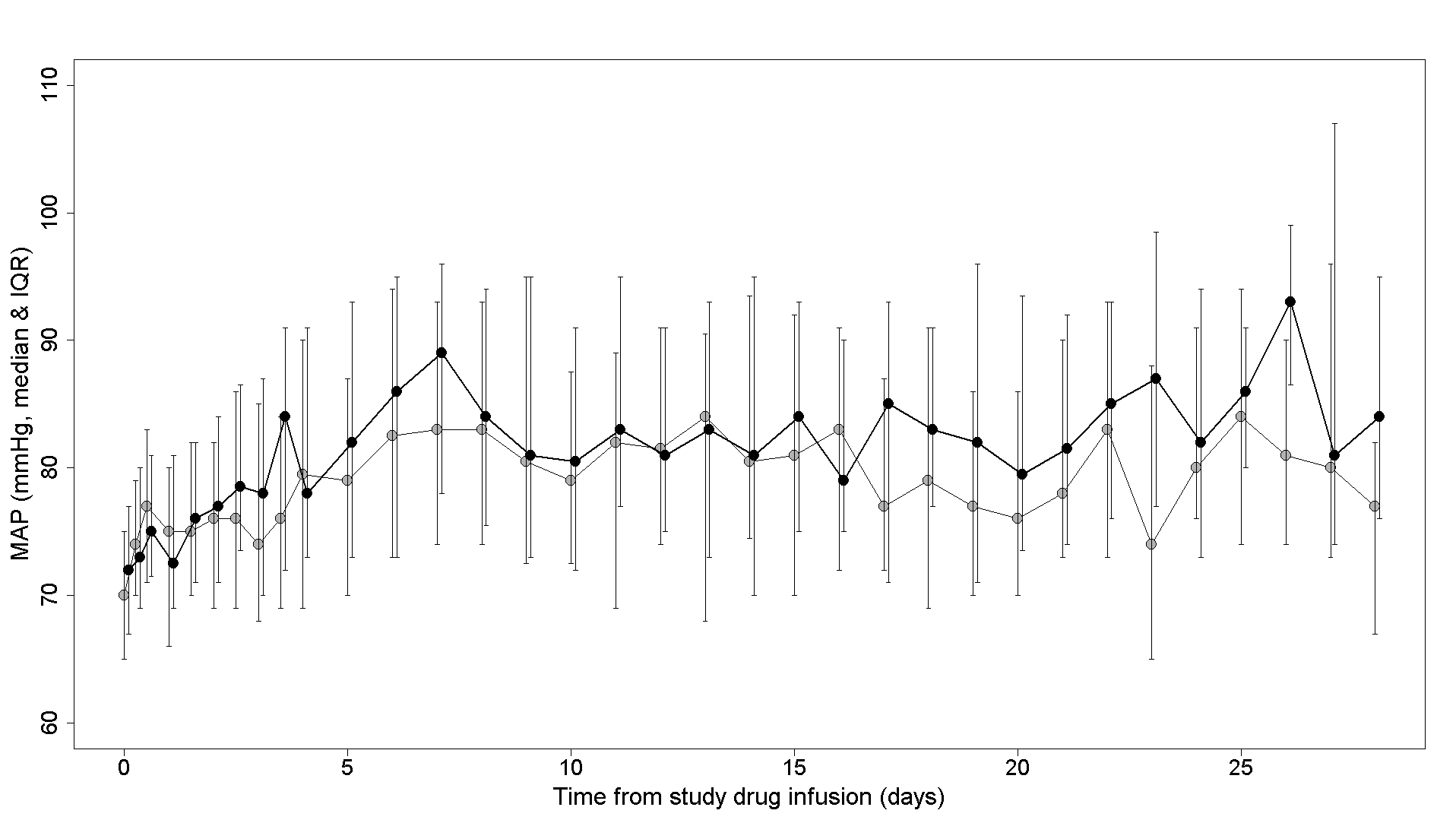


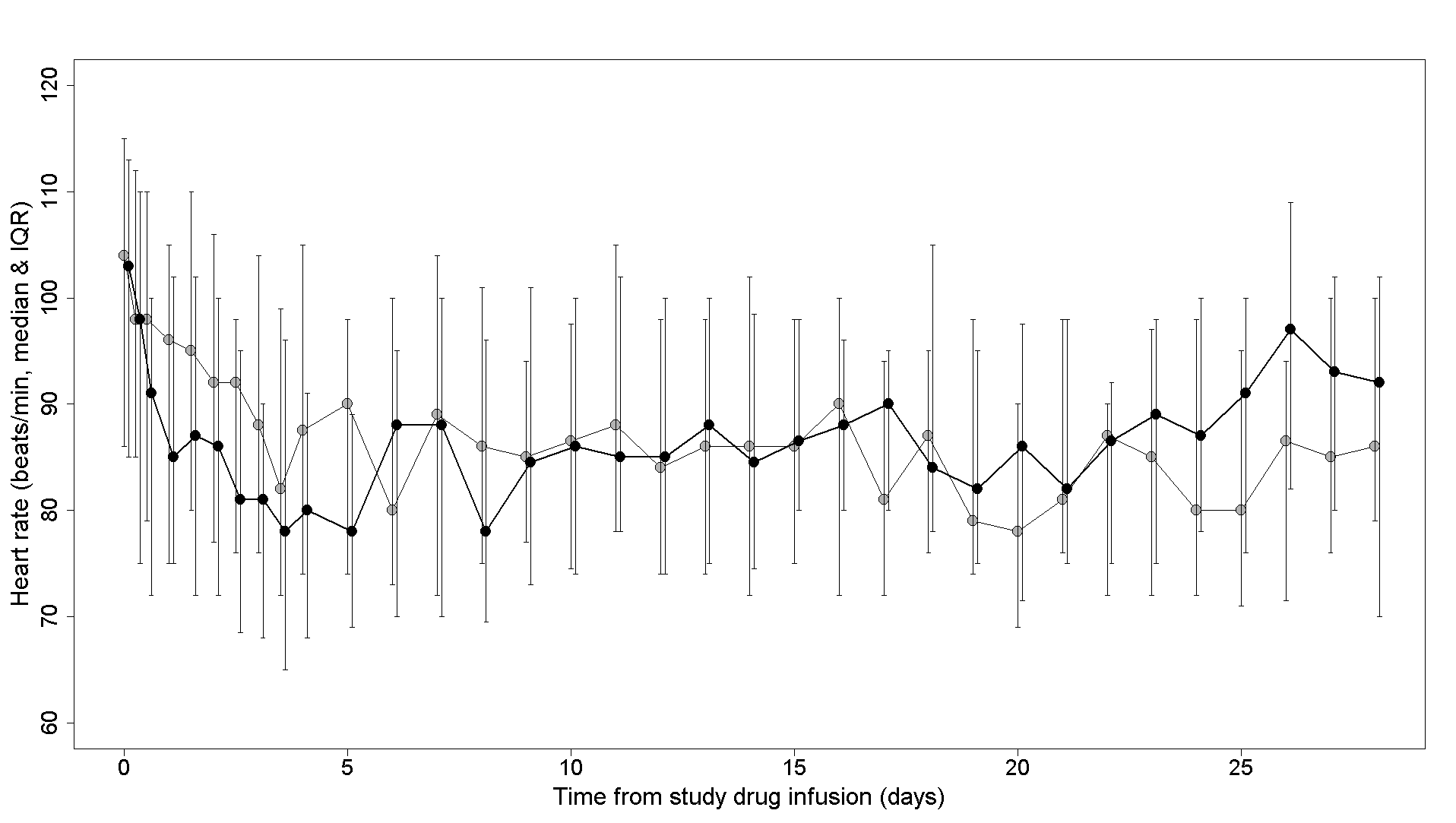


Additional file 5: Figure S1. Comparison of mean arterial pressure (above) and heart rate (below) in the norepinephrine group (grey lines) and vasopressin group (black lines). Values are median + interquartile range. There were no statistically significant differences between the norepinephrine and vasopressin groups in mean arterial pressure. Heart rate was significantly lower in the vasopressin group than in the norepinephrine group over the first four days (P=0.033).
