# Supplementary material for: Erratum to: Cardiac ischemia in patients with septic shock randomized to vasopressin or norepinephrine
Source: Crit Care. 2017 May 4;21:98. doi: 10.1186/s13054-017-1680-7 (PMC5415714; doi:10.1186/s13054-017-1680-7)
Supplement: Supplementary file 6 — Rates of total norepinephrine infusion (open-label and study drug) in the vasopressin treated group and the norepinephrine treated group. (DOCX 60 kb) [file 13054_2017_1680_MOESM6_ESM.docx]

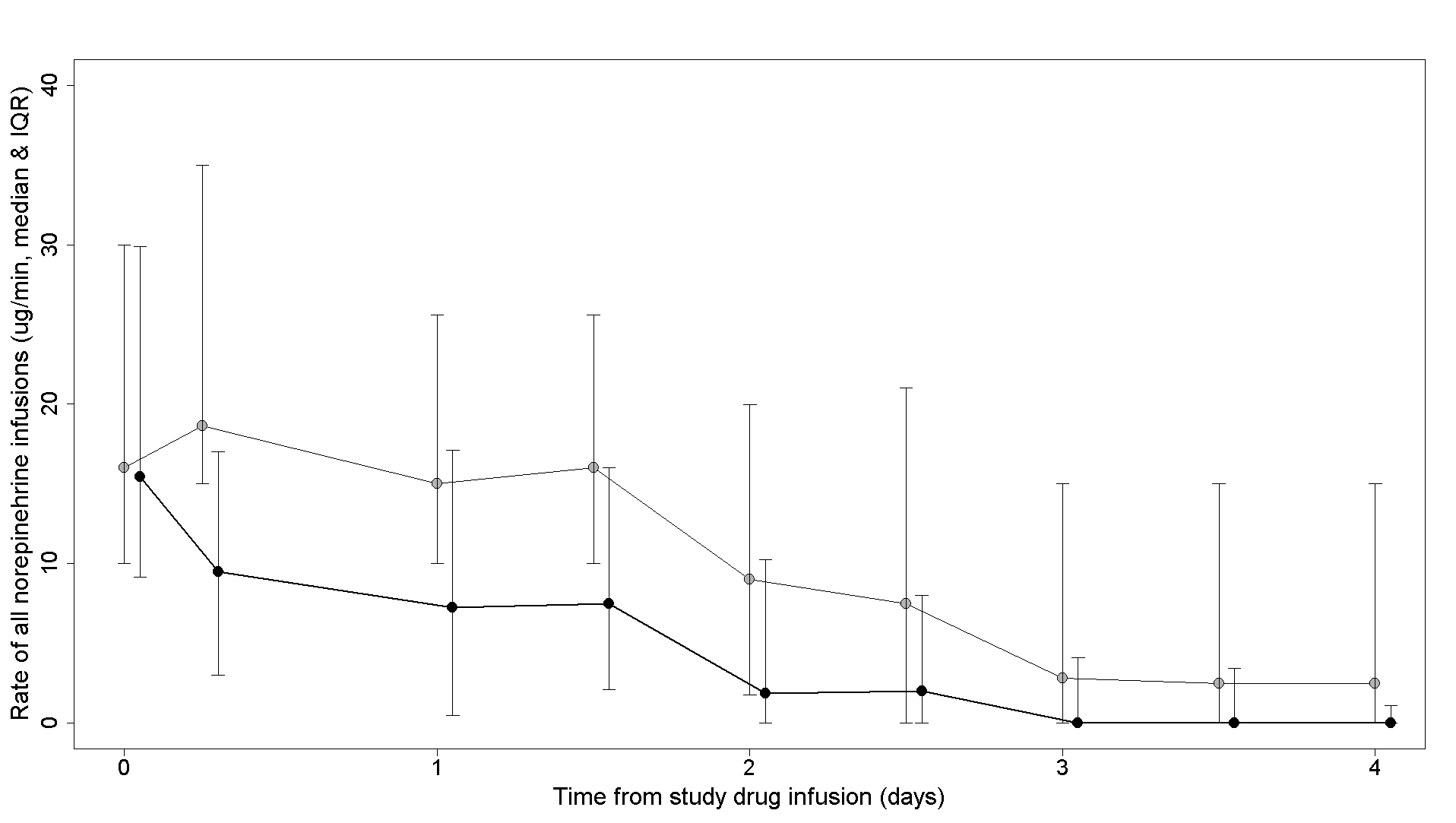


Additional file 6: Figure S2. Rates of total norepinephrine infusion (open-label and study drug) in the vasopressin treated group (black line) and the norepinephrine treated group (grey line) amongst patients who were treated only with open-label norepinephrine at baseline. The rates of norepinephrine infusion were significantly lower in the vasopressin group than in the norepinephrine group over the first four days (P=0.004). Values are median + interquartile range.
