# Supplementary material for: Erratum to: Cardiac ischemia in patients with septic shock randomized to vasopressin or norepinephrine
Source: Crit Care. 2017 May 4;21:98. doi: 10.1186/s13054-017-1680-7 (PMC5415714; doi:10.1186/s13054-017-1680-7)
Supplement: Supplementary file 7 — Serum creatinine in the vasopressin treated group and the norepinephrine treated group. (DOCX 64 kb) [file 13054_2017_1680_MOESM7_ESM.docx]

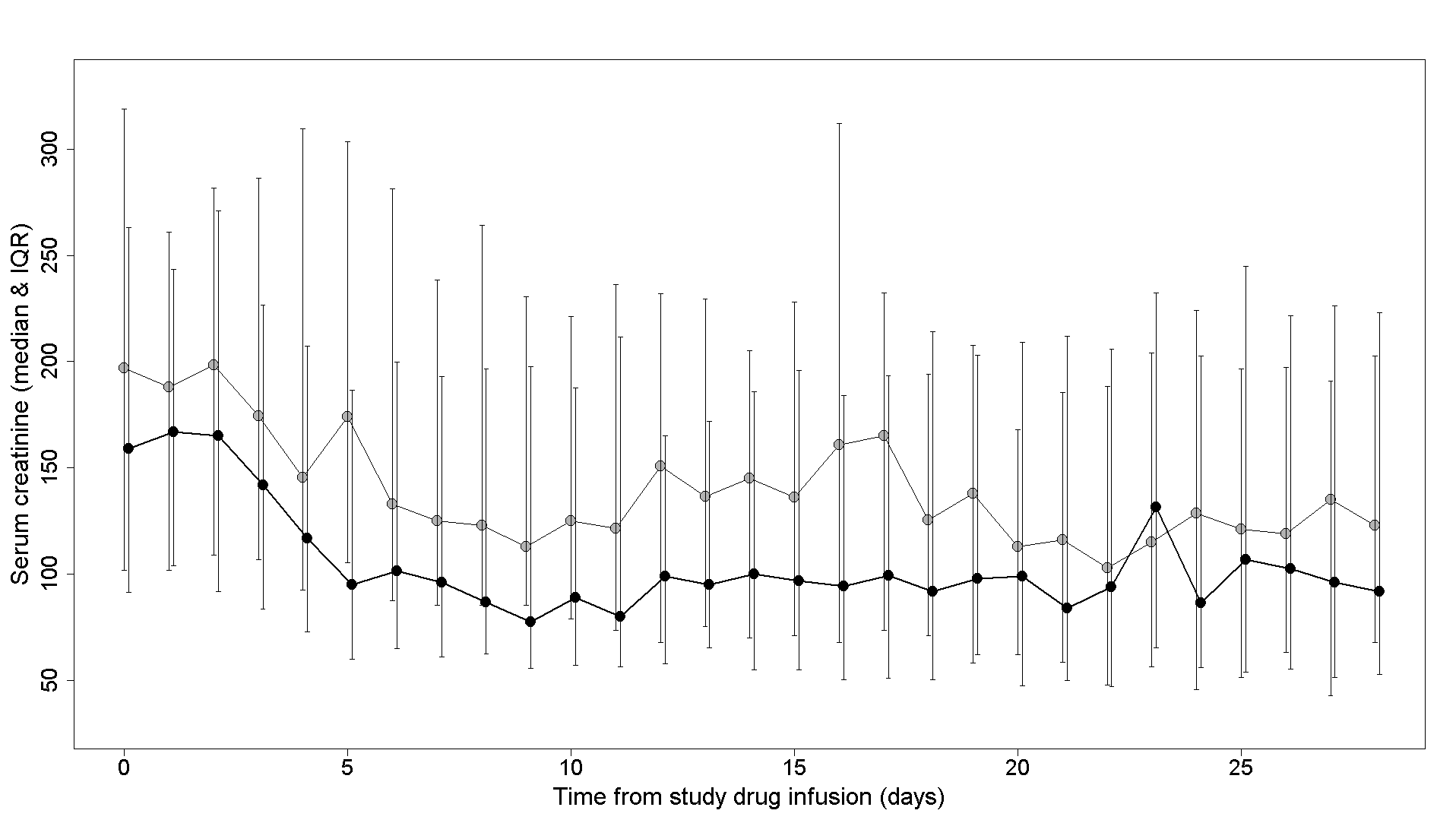


Additional file 7: Figure S3. Serum creatinine in the vasopressin treated group (black line) and the norepinephrine treated group (grey line). There were no statistically significant differences between the norepinephrine and vasopressin groups in serum creatinine. Values are median + interquartile range.
